# Evaluating Large Spontaneous Deletions in a Bovine Cell Line Selected for Bovine Viral Diarrhea Virus Resistance

**DOI:** 10.3390/v13112147

**Published:** 2021-10-25

**Authors:** Aspen M. Workman, Michael P. Heaton, Dennis A. Webster, Gregory P. Harhay, Theodore S. Kalbfleisch, Timothy P. L. Smith, Shollie M. Falkenberg, Daniel F. Carlson, Tad S. Sonstegard

**Affiliations:** 1 US Meat Animal Research Center, United States Department of Agriculture, Agricultural Research Service, Clay Center, NE 68901, USA; mike.heaton@usda.gov (M.P.H.); gregory.harhay@usda.gov (G.P.H.); tim.smith2@usda.gov (T.P.L.S.); 2 Recombinetics Inc., Eagan, MN 55121, USA; Dennis@recombinetics.com (D.A.W.); dan@recombinetics.com (D.F.C.); 3 Gluck Equine Research Center, Department of Veterinary Science, University of Kentucky, Lexington, KY 40506, USA; ted.kalbfleisch@uky.edu; 4 National Animal Disease Center, United States Department of Agriculture, Agricultural Research Service, Ames, IA 50010, USA; shollie.falkenberg@usda.gov; 5 Acceligen Inc., Eagan, MN 55121, USA; tad@acceligen.com

**Keywords:** BVDV, CRISPR, PTPN12, GRID2, RABGAP1L, CRIB, MDBK

## Abstract

Bovine viral diarrhea virus’s (BVDV) entry into bovine cells involves attachment of virions to cellular receptors, internalization, and pH-dependent fusion with endosomal membranes. The primary host receptor for BVDV is CD46; however, the complete set of host factors required for virus entry is unknown. The Madin-Darby bovine kidney (MDBK) cell line is susceptible to BVDV infection, while a derivative cell line (CRIB) is resistant at the level of virus entry. We performed complete genome sequencing of each to identify genomic variation underlying the resistant phenotype with the aim of identifying host factors essential for BVDV entry. Three large compound deletions in the BVDV-resistant CRIB cell line were identified and predicted to disrupt the function or expression of the genes *PTPN12*, *GRID2*, and *RABGAP1L*. However, CRISPR/Cas9 mediated knockout of these genes, individually or in combination, in the parental MDBK cell line did not impact virus entry or replication. Therefore, resistance to BVDV in the CRIB cell line is not due to the apparent spontaneous loss of *PTPN12*, *GRID2*, or *RABGAP1L* gene function. Identifying the functional cause of BVDV resistance in the CRIB cell line may require more detailed comparisons of the genomes and epigenomes.

## 1. Introduction

Bovine viral diarrhea virus (BVDV; family *Flaviviridae*, genus *pestivirus*) is one of the most widespread and economically important viral infections in cattle [1] with average direct losses approaching $175–200/head USD [2]. Acute infection results in systemic spread that is associated with respiratory and gastrointestinal diseases, immunosuppression, and reproductive failure [3,4]. BVDV can cross the placenta and infect the developing fetus resulting in abortion, malformations, or the birth of immunotolerant and persistently infected (PI) calves [5]. PI calves often have increased morbidity and mortality and are the most important sources of virus spread in the population [6]. Effective disease control relies on a combination of vaccination, identification and removal of PI calves, and implementation of biosecurity protocols [7]. However, diagnostic testing to identify PI cattle is labor intensive and costly and the genetic and antigenic variability of circulating field strains of BVDV pose challenges to the making of broadly protective vaccines [8]. Furthermore, BVDV infection of other wild and domestic ruminants is a potential threat to the success of BVDV control programs [4]. As a result, BVDV remains a significant pathogen globally. Development of novel intervention strategies could greatly improve animal welfare and virus control efforts while enhancing animal production.

The viral infection cycle begins when the virus binds and enters susceptible host cells. BVDV encodes three envelope glycoproteins that participate in entry: E^RNS^, E1, and E2. Interactions between E^RNS^ and negatively charged glycosaminoglycans, such as heparan sulfate, provide the first contact of the virus with the host cells and serves to concentrate the virus on the cell surface [9,10] and in some cases can facilitate virus entry [9,11,12]. Cellular CD46 has been identified as the primary cellular receptor for most BVDV isolates and is recognized by viral E2 [13,14]. Receptor binding triggers virus internalization by clathrin-dependent endocytosis [15,16]. As the endocytic vesicle matures, the combined action of low endosomal pH and disulfide bond reduction is proposed to mediate fusion between the virus and host membranes, resulting in the release of the viral nucleocapsid into the host cell cytoplasm [15]. Disassembly of the viral capsid (uncoating) delivers the viral RNA genome to the cytoplasm, completing the entry process. The complete set of host factors required for BVDV entry remains unknown; however, targeted disruption of any of these events could render cells resistant to infection. Elucidating critical virus-host interactions facilitating entry will not only advance our knowledge of pestivirus infection and pathogenesis but will also provide novel targets for intervention strategies.

Bovine kidney CRIB cells (i.e., Cells Resistant to Infection with BVDV) offer a unique opportunity to identify additional host factors required for pestivirus entry [17,18]. CRIB cells were originally isolated by cloning Madin-Darby bovine kidney (MDBK) cells that survived infection with the cytopathic Singer strain of BVDV [17]. Characterization of CRIB cells revealed they were resistant to infection by BVDV and other related pestiviruses due to a defect in virus entry [17,18], despite the normal expression of the cellular receptor CD46 on the cell surface [19]. Transfection of CRIB cells with BVDV RNA resulted in the production of infectious virus, revealing that the intracellular milieu is permissive to infection [17]. Together, these studies suggested that the entry of pestiviruses into MDBK cells depends on a common entry factor that is absent in CRIB cells. To date, the specific genomic changes that resulted in the resistant phenotype remain unknown. The goal of this study was to sequence the complete genomes of MDBK and CRIB cell lines to identify and evaluate any major genomic changes that could be responsible for the pestivirus-resistant phenotype of CRIB cells.

## 2. Materials and Methods

### 2.1. Cell Lines and Viruses

BVDV-free Madin-Darby bovine kidney cells (MDBK; ATCC CCL-22, Lot no. 3752721, passage 113) and CRIB cells (i.e., “Cells Resistant to Infection with BVDV”, ATCC CRL-11883, Lot no. 100005, Reference CRIB1P35, passage 35, [17]) were obtained from the American Type Culture Collection (ATCC; Rockville, MD, USA). Individual and triple gene knockout (KO) clones were made in MDBK cells as described below. Cells were maintained in minimum essential media (MEM; Gibco, Grand Island, NY, USA) supplemented with 10% irradiated fetal bovine serum (Atlas Biologicals, Fort Collins, CO, USA), 1× antibiotic-antimycotic (Gibco) and 2 mM L-Glutamine (Gibco) under 5% CO_2_ at 37 °C. Cell growth rate was determined by plating 2.5 × 10^5^ cells in triplicate in T-25 flasks. Cells were trypsinized and counted using a Countess II automated cell counter (Invitrogen; Carlsbad, CA, USA) every 24 h for 96 h. Cell numbers were graphed in Prism, version 6 (GraphPad Software, San Diego, CA, USA). Cells were imaged using the EVOS FL auto microscope (Invitrogen).

Cytopathic BVDV-1a strain NADL (ATCC-VR1422) was purchased from ATCC. Cytopathic BVDV strains Singer (BVDV-1a), MDA280n (BVDV-1b), IIICPE (BVDV-1b), TGAC (BVDV-1b), 53637 (BVDV-2), and 296c (BVDV-2) were from the National Animal Disease Center (NADC) collection in Ames, Iowa. Cytopathic BVDV strains were propagated in MDBK cells and quantitated on bovine turbinate cells (BT; ATCC CRL 1390). The infective titer was determined in two replicates using an endpoint dilution assay. Viral stocks were stored in 0.5 to 1 mL aliquots at −80 °C.

Non-cytopathic BVDV-2 isolate PI-92-2014 was isolated on MDBK cells from the plasma of a calf persistently infected with BVDV [20]. The virus was passaged four times on MDBK cells and a viral stock was prepared. The infective titer was estimated by RT-qPCR using log_10_ dilutions of the virus. A standard curve was made by plotting RT-qPCR Ct values against log_10_ dilutions of the NADL virus with a known infectious titer. Linear regression analysis was performed to create a standard for estimating the titer of non-cytopathic stocks of virus. BVDV isolates PI-31-2019 (BVDV-1a), PI-60-2019 (BVDV-1b), PI-64-2019 (BVDV-1b), and PI-65-2019 (BVDV-1b) were tested by directly inoculating cells with serum obtained from calves persistently infected with BVDV (unpublished virus isolates).

### 2.2. Whole Genome Sequence (WGS)

The WGS of MDBK, CRIB, and KO clones was accomplished with methods as described elsewhere [21]. Briefly, genomic DNA was used to make a 500 bp paired-end library (TruSeq DNA PCR-Free kits, Illumina, San Diego, CA, USA) and sequenced on a NextSeq500 (two by 150 paired-end reads, Illumina) until a minimum of 40 GB of data with greater than Q20 quality was collected. The raw reads were filtered to remove adaptor sequences, contaminating dimer sequences, and low-quality reads. The DNA sequence alignment process was similar to a previous report [21]. FASTQ files were aggregated for each sample and DNA sequences were aligned individually to the bovine reference assembly ARS-UCD1.2 ([22] Accession ID: GCF_002263795.1. NCBI Genome ID: 82; Bos Taurus) with the Burrows-Wheeler aligner (BWA) aln algorithm version 0.7.12, then merged and collated with bwa sampe. The resulting sequence alignment map (SAM) files were converted to binary alignment map (BAM) files, and subsequently sorted via SAMtools version 0.1.18 [23]. Potential PCR duplicates were marked in the BAM files using the Genome Analysis Toolkit (GATK) version 1.5-32-g2761da9 [24]. Regions in the mapped dataset with small indels that would benefit from re-alignment were identified with the GATK module RealignerTargetCreator and realigned using the module IndelRealigner. The BAM files produced at each of these steps were indexed using SAMtools. The raw reads were deposited at NCBI BioProject PRJNA761701.

Deletions in the genome of the CRIB cell line relative to MDBK cells were identified with bedtools 2.27.1 to complete genomic interval comparisons and read map file manipulations as detailed on this internet site for protocol sharing (link: https://www.protocols.io/view/evaluating-large-spontaneous-deletions-in-a-bovin-bvv3n68n, accessed on 20 October 2021). Bedtools bamtobed was used to convert each cell line’s BAM file into a BED file. Using the bedtools intersect command, reads within a cell line’s BED that intersected with repeat regions in the RepeatMasked ARS-UCD1.2 bovine genome were removed to create a RepeatMasked BED file. Using the RepeatMasked BED files, the bedtools intersect command was used to remove reads in the MDBK library that overlapped with reads in the CRIB library to create a BED file of reads unique to the MDBK genome. This BED file of MDBK cell reads mapping to regions of the genome not covered by reads from CRIB (BED file named “MDBK+_CRIB-.bed”) was manually screened with the Integrative Genomics Viewer (IGV, v2.9.4), identifying three read-rich regions not populated in CRIB (Figure S1).

### 2.3. Annotation of RABGAP1L with Long-Read RNA Sequence Data

Ambiguity surrounding the annotation and genome assembly in the *RABGAP1L* gene region was clarified by first determining the *RABGAP1L* RNA isoforms expressed in cattle using full-length RNA transcript sequences produced from single-molecule, real-time sequencing (Iso-Seq method, Pacific Biosciences platform, access date 12 March 2018). The Hereford, Angus, and Brahman full-length RNA transcripts are available at NCBI under BioProjects PRJNA386670 and PRJNA432857, respectively. These sequences were searched by blastn using the RefSeq transcript for *RABGAP1L* (accession NM_001103263, access date 12 March 2018) as the query sequence. Sequences with pairwise identity >95% were selected and used for manual annotation of *RABGAP1L* on the region between 55,479,000 and 56,227,501 of chromosome 16 extracted from the ARS-UCD1.2 assembly in GenBank (accession NC_037343.1, access date 12 March 2018). Transcript sequences from other mammals were also aligned to this region to evaluate conservation at this locus.

### 2.4. RNA Sequencing

MDBK and CRIB cells were grown in triplicate to 90% confluency in 6-well culture plates (yielding approximately 1 × 10^6^ cells per well). Total RNA was isolated from cells in each well using TRIzol (Life Technologies, Carlsbad, CA, USA) according to the manufacturer’s instructions. Genomic DNA contamination was removed using the Turbo DNA-free kit (Thermo Fisher Scientific, Waltham, MA, USA). RNA integrity and concentration were quantified with a 5200 Fragment Analyzer System (Agilent Technologies, Santa Clara, CA, USA). An Illumina TruSeq stranded mRNA library preparation kit was used to construct cDNA libraries, which were sequenced as 75-bp paired-end reads on an Illumina NextSeq 500 (Illumina, San Diego, CA, USA) to the depth of approximately 25 million reads per library.

The sequence data were processed within the OmicsBox environment (BioBam Bioinformatics V.1.4.11, 3 March 2019). Read quality metrics were assessed with FastQC. All libraries passed quality checks for Per Base Sequence Quality, Per Sequence Quality Scores, Per Base N Content, and Adapter Content. The reads were then trimmed with Trimmomatic (v. 0.38) to remove TruSeq3-SE adapters with sliding window trimming with a 4 bp window and 20 minimum quality. The final trimmed reads were required to have an average quality score of at least 25 with a minimum length of 36 bp. The filtered & trimmed output was aligned to the ARS-UCD1.2 reference bovine genome with the STAR aligner [25] using two-pass mapping, a minimum intron length of 20 bp, a maximum intron length and distance between mates of 1,000,000 bp, a maximum number of multiple alignments of 20, a maximum number of mismatches of 999, and chimeric reads were excluded from the alignment. HTSeq (v. 0.9.0 [26]) was used to count aligned sequencing reads with the quantification level set to gene, with overlap mode set to union, strand-specific switch set to reverse, and with a minimum mapping quality of 10. Pairwise differential expression analyses between MDBK and CRIB libraries were conducted with edgeR [26,27] with libraries normalized using the trimmed mean of M values method (TMM) [28].

### 2.5. Generation of MDBK Single and Triple Knockout (KO) Clones

#### 2.5.1. gRNA Design and Production

Candidate gRNAs were designed using Cas-Designer [29] and construction oligos were purchased from Integrated DNA Technologies (IDT; San Jose, CA, USA). Oligos were annealed by heating to 95 °C for 5 min and cooling to room temperature over a span of one hour. The gRNA transcription vector (pDR274 for btGRID2 and RABGAP1L or pDR274-SP6 for PTPN12) was linearized with BsaI (New England Biolabs, NEB; Ipswich, MA, USA) and purified using the QIAquick Gel Extraction Kit (Qiagen, Hilden, Germany). The annealed oligos were ligated into the linearized transcription vector using T4 DNA Ligase (NEB) and transformed into TOP10 (Invitrogen, Waltham, MA, USA) competent cells. Two milliliters of 2xYT media supplemented with kanamycin was inoculated with a single clone and grown overnight at 37 °C. The plasmid was purified from the overnight culture using the Qiagen Miniprep Kit. Transcription plasmids were linearized with DraI (New England Biolabs) and amplified using Accustart Taq DNA Polymerase HiFi (Quanta Biosciences, Gaithersburg, MD, USA) using the following primers and cycling program: pDR274 F (5′-TCCGCTCGC-ACCGCTAGCT-3′) and pDR274 R (5′-AGCACCGACTCGGTGCCAC-3′); 1 cycle (95 °C, 2 min), 35 cycles of (95 °C, 30 s; 48 °C, 15 s; 68 °C, 15 s), 1 cycle (68 °C, 30 s). Once completed, the amplification reactions were treated with RNAsecure (Thermo Fisher Scientific) following the manufacturer’s recommendations and purified using the QIAquick Gel Extraction Kit (Qiagen). These amplicons were used as template for transcription using the MEGAshortscript T7 Transcription Kit (Thermo Fisher Scientific) for GRID2 and RABGAP1L or HiScribe SP6 RNA Synthesis Kit (NEB) for PTPN12 and purified with the RNeasy Mini Kit (Qiagen), following manufacturer’s instructions.

#### 2.5.2. Cas9 mRNA Preparation

pT3Ts-nCas9n plasmid was linearized with XbaI (NEB), treated with RNAsecure (Thermo Fisher Scientific) and purified using the QIAquick Gel Extraction Kit (Qiagen). In vitro Cas9 mRNA transcription was performed using the mMESSAGE mMACHINE T3 Kit (Thermo Fisher Scientific) followed by A-tailing using a Poly(A) Tailing Kit (Thermo Fisher Scientific). These transcripts were purified with the RNeasy Mini Kit (Qiagen), following manufacturer’s instructions.

#### 2.5.3. Tissue Culture and Transfection for Single KO Clones

MDBK cells were briefly maintained at 38.5 °C at 5% CO_2_ in DMEM supplemented with 10% fetal bovine serum, 100 I.U./mL penicillin and streptomycin, 2 mM L-Glutamine and 10 mM Hepes. Once the cells reached 80% confluency, they were spilt 1:2 and harvested the next day at 70–80% confluency. Approximately 600,000 cells were suspended in “R” Buffer (Life Technologies) and electroporated with the Neon Transfection system (Life Technologies) using the 100 uL tips and the following parameters: input voltage: 1600 V; pulse width: 20 ms; pulse number: 1. The transfection system delivered 1 μg of the appropriate gRNA(s), 2 μg nCas9n mRNA per gRNA, and 0.2 nMol of the appropriate single-stranded oligo donor (ssODN). Guide RNAs and ssODN are listed in Table 1. Transfected cells were dispersed into one well of a 6-well plate with 2 mL DMEM media and cultured for three days at 30 °C.

#### 2.5.4. Single-Cell Derived Clonal Isolation and Genotyping

Four days after transfection, cells were seeded onto 10 cm plates at a density of 30 cells/plate and cultured until individual colonies reached approximately 5 mm in diameter. The colonies were aspirated and replicate plated on 48- and 96-well plates. The 96-well plates were incubated for 2 days prior to lysis. Cells were resuspended in 20 μL of 1X PCR compatible lysis buffer (10 mM Tris-Cl pH 8.0, 2 mM EDTA, 0.45% Triton X-100 (*v/v*), 0.45% Tween-20 (*v/v*)) freshly supplemented with 200 μg/mL Proteinase K. The lysates were incubated in a thermal cycler using the following program: 55 °C for 60 min, 95 °C for 15 min.

#### 2.5.5. Mutation Detection

Identification of PTPN12 and *GRID2* KO was performed by PCR amplification (AccuStart II GelTrack PCR SuperMix, Quanta Biosciences, Beverly, MA, USA) with 1 μL of the cell lysate as template using the following primers and cycling program: PTPN12 NJ F1 (5′-ACCATGTTTCACCAGAAGCTGA-3′) and PTPN12 NJ R1 (5′-ACAAGACAACCTAGCCATCAGT-3′); or GRID2 NJ F1 (5′-ACTTGGATGTGGGCTAAGCA-3′) and GRID2 NJ R1 (5′-CTGAATGCAATGCCACCAGA-3′); 1 cycle (95 °C, 2 min), 35 cycles of (95 °C, 20 s; 60 °C, 20 s; 72 °C, 45 s), 1 cycle (72 °C, 5 min). Amplicons were then cut with *HindIII* (NEB) and visualized using agarose gel electrophoresis. Clones homozygous by RFLP for *GRID2* and *PTPN12* KO alleles were verified by Sanger sequencing (ACGT, Germantown, MD, USA).

Identification of the *RABGAP1L* deletion was performed by PCR amplification as described above with 1 μL of the cell lysate as template using the following primers and cycling program: RABGAP1L 5′ NJ F1 (5′-GCCCAGCAATCCCTCTTTTG-3′) and RABGAP1L 3′ NJ R1 (5′- ACCTTTCATCTCTTGTGCCC-3′); 1 cycle (95 °C, 2 min), 35 cycles of (95 °C, 20 s; 60 °C, 20 s; 72 °C, 45 s), 1 cycle (72 °C, 5 min). PCR amplification of the 5′ junction was conducted as described above with 1 μL of the cell lysate as template using the following primers and cycling program: RABGAP1L 5′ NJ F1 (5′-GCCCAGCAA-TCCCTCTTTTG-3′) and RABGAP1L 5′ NJ R1 (5′-AGACCCCTGGATCAGTGGTT-3′); 1 cycle (95 °C, 2 min), 35 cycles of (95 °C, 20 s; 60 °C, 20 s; 72 °C, 45 s), 1 cycle (72 °C, 5 min). PCR amplification of the 3′ junction was conducted as described above with 1 μL of the cell lysate as template using the following primers and cycling program: RABGAP1L 3′ NJ F1 (5′- AACCAAGTTGAATTTTGCAGTG-3′) and RABGAP1L 3′ NJ R1 (5′- ACCTTTCATCTCTTGTGCCC-3′); 1 cycle (95 °C, 2 min), 35 cycles of (95 °C, 20 s; 60 °C, 20 s; 72 °C, 45 s), 1 cycle (72 °C, 5 min).

#### 2.5.6. Selection of Clones for Downstream Testing

Two Sanger-sequence confirmed clones with similar growth rates and morphological characteristics to the parental MDBK cell line were selected for virus susceptibility testing. One KO clone from each gene of interest was further selected for WGS as described above.

#### 2.5.7. Generation of Triple Gene KO

One colony that was homozygous for the RABGAP1L KO was expanded and used as the parental line for the creation of the triple KO cell line. This clone was transfected as described above using the Neon transfection system (Life Technologies) to deliver 1 ug GRID2 gRNA, 1 ug PTPN12 gRNA, 4 ug nCas9n mRNA, 0.2 nmol GRID2 g2 HD3-KO ssODN and 0.2 nMol PTPN12 g3 HD3-KO ssODN (Table 1). Single-cell derived clones were screened for desired mutations as described above. One clone was selected for virus susceptibility testing and WGS as described above.

### 2.6. SDS-PAGE and Immunoblotting

Whole-cell lysates were prepared from cells grown in 6-well culture plates for 48 h. Cells were washed with phosphate-buffered saline (PBS) and suspended in RIPA lysis buffer [150 mM sodium chloride, 1.0% Triton X-100, 0.5% Deoxycholic Acid, 0.1% SDS, 50 mM Tris, pH 8.0]. Cell lysate was incubated on ice for 30 min, sonicated, and then clarified by centrifugation at 18,000× *g* at 4 °C for 5 min. Protein concentrations were quantified using a Bicinchoninic Acid (BCA) protein assay kit (Thermo Fisher Scientific). Samples were diluted to 40 μg in a commercial sample buffer with lithium dodecyl sulfate and dithiothreitol and used per the manufacturer’s instructions (Thermo Fisher Scientific). Samples were heated to 70 °C for 10 min and loaded on 4–12% precast polyacrylamide Bis-Tris Plus gels (Thermo Fisher Scientific) at run 160 volts for approximately 45 min in 2-(N-morpholino)ethane-sulfonic acid (MES) SDS running buffer (Thermo Fisher Scientific). After electrophoresis, proteins were transferred onto a polyvinylidene difluoride membrane (Immobilon-P; Millipore) and nonspecific antibody binding to the membrane was blocked by incubating 2 h in 5% nonfat dry milk (NFDM) or 2% bovine serum albumin (BSA) in Tris-buffered saline-0.1% Tween 20 (TBS-T) depending on the antibody used (see details below). Membranes were then incubated with primary antibody overnight at 4 °C with gentle agitation. The antibodies used were anti-GRID2 (Abcam, Cambridge, United Kingdom, catalog no. ab190358) at a 1:1000 dilution in 2% BSA in TBS-T; anti-PTPN12 (Thermo Fisher Scientific, catalog no. PA5-27733) diluted 1:5000 in 5% NFDM in TBS-T; and anti-GAPDH (Abcam catalog no. ab181603) diluted 1:10,000 in 5% NFDM in TST-T. The following day, membranes were washed three times for 10 min each with TBS-T then incubated with goat anti-rabbit HRP secondary antibody (Thermo Fisher Scientific catalog no. 32460) at a 1:1000 dilution in the same buffer as the primary antibody for 1 hr at room temperature. Blots were then washed three times for 10 min each with TBS-T prior to detection. For detection, the immunoblot was incubated in a chemiluminescent substrate (Amersham ECL Prime, GE Healthcare) for 5 min and imaged (ChemiDoc, Bio-Rad Laboratories, Inc. Hercules, CA, USA).

### 2.7. Multistep Virus Growth Curves

Cells were seeded in 48-well plates at a density of 5 × 10^4^ cells per well in 200 µL MEM supplemented with 1x antibiotic-antimycotic (Gibco brand, Thermo Fisher Scientific) and 2 mM L-Glutamine (Gibco), and 3.75% horse serum (ATCC, cat no. ATCC 30-2040) and incubated 24 h at 37 °C with 5% CO_2_. Virus was then added to plates at an MOI of 0.01 in 100 µL MEM supplemented with 1x antibiotic-antimycotic and 2 mM L-Glutamine, bringing the final horse serum concentration to 2.5%. An input plate (t = 0 h post-infection) was frozen at −80 °C immediately after infection and the remaining plates were incubated at 37 °C with 5% CO_2_ for 24, 48, 72 or 96 h. Plates were frozen at −80 °C at the indicated time post-infection. Plates were thawed at 37 °C and cells were collected and transferred to a 1.5 mL microcentrifuge tube, vortexed, and frozen at −80 °C. Cell were thawed a second time, vortexed, and centrifuged at 18,000× *g* for three minutes at 4 °C to pellet cellular debris. The supernatant was transferred to a new 1.5 mL microcentrifuge tube and stored at −80 °C until processed for RNA. Trizol LS (Life Technologies) was used to extract RNA per the manufacturer’s instructions. Viral RNA was quantified by RT-qPCR with a BVDV specific primer and probe set [30]. Cyclic amplification reactions were carried out in a 25 μL reaction containing: 4.5 mM MgCl_2,_ 400 μM each dNTP, 0.4 μM of each primer, 0.2 μM probe, 1 μL enzyme mix containing reverse transcriptase (RT) and a hot start Taq polymerase (OneStep RT-PCR kit, Qiagen Inc., Venlo, The Netherlands), and 5 μL RNA. Cycling conditions were as follows: reverse transcription for 30 min at 50 °C, inactivation of RT enzyme and activation of Taq polymerase for 15 min at 95 °C followed by 40 cycles of 94 °C for 30 sec, 55 °C for 60 sec and 72 °C for 60 s. Cycle threshold (Ct) values less than 38 were considered positive. Positive, negative, no template, and extraction controls were included on each run. The fold increase in viral RNA compared to the input concentration was determined with the delta Ct method [31] and graphed in Prism, version 6 (GraphPad Software, San Diego, CA, USA).

## 3. Results

### 3.1. Identification of Three Large Genomic Deletions in the BVDV-Resistant CRIB Cell Line

**Figure 1:**
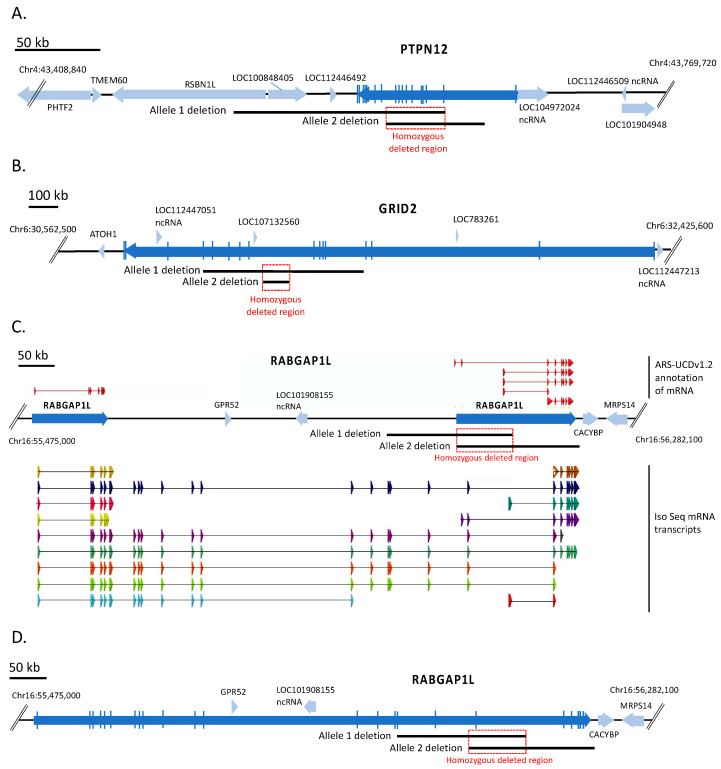
Physical maps of the three large homozygous deleted regions in the CRIB cell line. Genome map of ARS-UCD1.2 in the region of the three large homozygous deleted regions. Blue arrows, genes (open reading frames); black lines, overlapping heterozygous deletion alleles; red box, homozygous deleted region. Panel (**A**) heterozygous deletion alleles of *PTPN12* on chromosome 4. Panel (**B**) heterozygous deletion alleles of *GRID2* on chromosome 6. Panel (**C**) heterozygous deletion alleles of *RABGAP1L* on chromosome 16. Shown on top of the bovine map are the transcripts annotated for *RABGAP1L* in the Hereford ARS-UCD1.2 reference genome. Below the map are IsoSeq transcripts from multiple cattle breeds (see Methods). Panel (**D**), genome map with the full-length bovine *RABGAP1L* gene as determined using IsoSeq transcripts.

Propagation of cell lines frequently results in chromosomal deletions and rearrangements that represent potential candidates for the loss of viral susceptibility observed in the CRIB cell line. Therefore, our first analysis focused on identifying homozygous deleted regions present in the CRIB cell line compared to the MDBK parent line since these could potentially cause the altered phenotype. Whole genome sequence of MDBK (48 Gb) and CRIB (62 Gb) cells was generated and aligned to the bovine reference assembly ARS-UCD1.2, with an average genome coverage of approximately 17- and 22-fold, respectively based on a 2.8 Gb genome. Using open-source software and custom scripts, major homozygous deleted regions of the CRIB cell line were identified by comparing the mapped read density between WGS libraries of CRIB and MDBK cell lines (see Methods Section 2.2 Whole genome sequencing, Figure S1). Three homozygous deleted regions were identified on three different chromosomes, each spanning more than 1 kb (Table S1). Manual inspection of these regions aligned to the bovine reference assembly revealed that all three regions consist of compound heterozygous deletions. These occur where a deletion on one copy of the chromosome overlaps a different deleted region on the other copy of the chromosome to produce a smaller region of homozygous loss. The affected regions were on chromosomes 4, 6, and 16 and were predicted to disrupt the genes *PTPN12*, *GRID2*, and *RABGAP1L*, respectively (Figure 1).

The *PTPN12* gene on chromosome 4 encodes the protein tyrosine phosphatase non-receptor type 12 protein. The CRIB cell line *PTPN12* gene contained a 57,648 bp deletion on one chromosome spanning exons 2–11, and a 123,042 bp deletion spanning exons 3–18 on the other chromosome. These heterozygous deletions overlapped resulting in a 33,808 bp homozygous deleted region predicted to remove exons 3–11 of *PTPN12* (Figure 1A) and together suggest that no functional protein would be produced in CRIB cells.

The *GRID2* gene on chromosome 6 encodes the glutamate ionotropic receptor delta type subunit 2 protein. The parental MDBK cell line contained a 472,427 bp deletion on one chromosome that spanned exons 5–12 of *GRID2.* The CRIB cell line acquired a nested deletion of 63,200 bp on the other chromosome resulting in a homozygous deleted region of 63,200 bp within intron 8 of *GRID2* (Figure 1B). Thus, at least one copy of the gene is unlikely to produce a functional protein, while the homozygous deletion in the intron would have unknown effects on protein structure or abundance.

The *RABGAP1L* gene on chromosome 16 encodes the RAB GTPase activating protein 1 like protein. Annotation of this gene in the bovine reference assembly (ARS-UCD1.2) includes two adjacent loci labeled as *RABGAP1L* in the same transcriptional direction (Figure 1C). The two loci do not share substantial sequence homology, and thus do not appear to be evidence of a gene duplication. Rather, analysis of other mammalian transcripts from this gene along with long read isoform sequencing of cattle tissues indicates the presence of multiple isoforms as illustrated in Figure 1C, some of which appear to be restricted to one or the other annotated gene, but most of which encompass both annotated loci. This indicated that the annotation is in error and a single *RABGAP1L* gene lies at this position of chromosome 16. Using this single *RABGAP1L* gene annotation model consistent with other mammalian species, one copy of the gene in CRIB cells has a 169,390 bp deletion removing exons 17–19, and the other copy has a 161,794 bp overlapping deletion removing exons 19–26. The overlap defines a 69,384 bp homozygous deletion that includes exon 19 (Figure 1D). Neither allele would be expected to produce a functional full-length protein. The biological significance of shorter transcripts containing only upstream or downstream exons is unknown.

Cellular localization, expression, and functions for the three genes have been reported in other mammals (Table 2). Research documenting a role for these genes in virus entry has not been reported; however, roles in endocytosis or cell signaling could be relevant to the observed phenotype in CRIB cells.

### 3.2. Loss of PTPN12 Protein Expression Does Not Significantly Impact BVDV Infection in MDBK Cells

**Figure 2:**
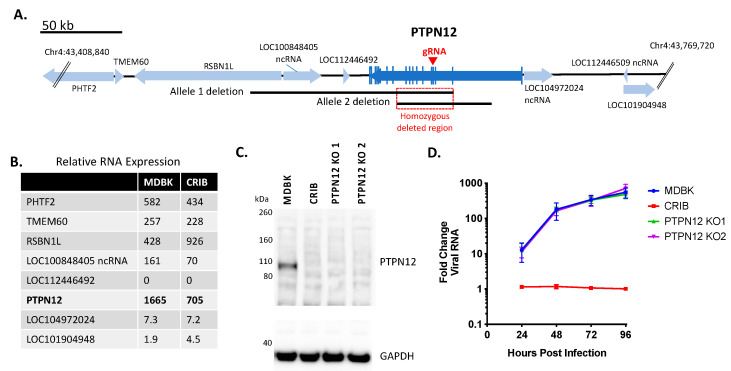
Knockout of PTPN12 does not significantly impact BVDV infection in MDBK cells. Panel (**A**), map of bovine *PTPN12* and surrounding genes: blue arrows, genes (open reading frames); vertical blue lines, coding regions (exons); black lines, heterozygous deletions in two alleles of *PTPN12* on chromosome 4; red square, homozygous deleted region (33,808 bp); red arrow, exon target of *PTPN12* gRNA. Panel (**B**), RNASeq analyses of relative RNA transcript abundance in MDBK and CRIB cells (trimmed mean of M (TMM) normalization value). Panel (**C**), western blot of SDS-PAGE for PTPN12 and GAPDH (loading control). Panel (**D**), Multistep virus growth curve. Cells were infected with BVDV strain NADL (MOI 0.01). Cells were collected and processed 0–96 h post-infection for quantitation of viral RNA using RT-qPCR. Results represent the mean ± standard deviation (*n* = 3).

The spontaneous deletions on chromosome 4 in CRIB cells were first examined for their potential effects on the relative mRNA transcript abundance of *PTPN12* and surrounding genes compared to MDBK (Figure 2A,B). The abundance of *PTPN12* mRNA and adjacent LOC100848405 ncRNA was reduced by 58% and 57%, respectively, in the CRIB cells compared to MDBK. Conversely, the adjacent RSBN1L RNA expression was two-fold higher in CRIB cells compared to MDBK cells. The abundance of RNA transcripts from four other genes in the area were approximately the same between cell lines.

The potential for the deletions within the CRIB *PTPN12* gene to interfere with viral entry or replication was directly tested by creation of MDBK *PTPN12* KO clones. These were generated by the introduction of a premature termination codon and a frameshift mutation in exon 4 using CRISPR/Cas9 technology to eliminate all known splice variants (Figure 2A). PTPN12 protein expression was assessed by an anti-PTPN12 immunoblot, which confirmed loss of PTPN12 protein expression in CRIB cells and PTPN12 KO clones 1 and 2 (Figure 2C). To determine whether loss of PTPN12 impacts virus replication kinetics, MDBK, CRIB, and *PTPN12* KO cells were infected with BVDV at a low MOI and viral RNA replication was monitored over time. Loss of PTPN12 did not significantly affect viral replication kinetics in MDBK cells (Figure 2D). Thus, disruption of *PTPN12* alone was not sufficient to affect BVDV infection of MDBK cells.

### 3.3. Loss of GRID2 Protein Expression Does Not Significantly Impact BVDV Infection in MDBK Cells

**Figure 3:**
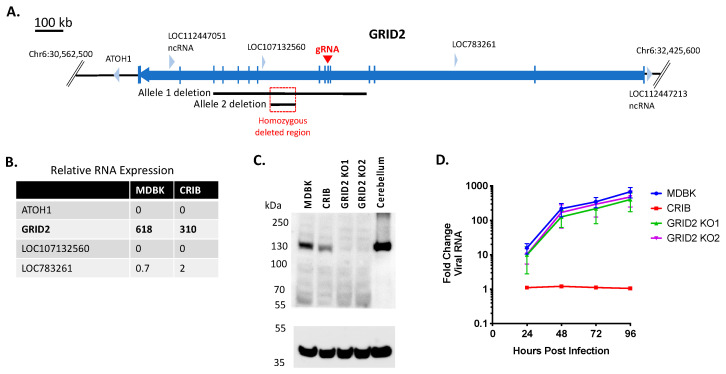
Knockout of GRID2 does not significantly impact BVDV infection in MDBK cells. Panel (**A**) map of bovine *GRID2* and surrounding genes: blue arrows, genes (open reading frames); vertical blue lines, coding regions (exons); black lines, heterozygous deletions in two alleles of *GRID2* on chromosome 6; red square, homozygous deleted region (63,200 bp); red arrow, exon target of *GRID2* gRNA. Panel (**B**) Relative RNA expression levels in MDBK and CRIB cells (TMM normalization value). Pane (**C**) western blot of SDS-PAGE for GRID2 and GAPDH (loading control). Bovine cerebellum was used as a positive control for GRID2 expression. Panel (**D**) Multistep virus growth curve. Cells were infected with BVDV strain NADL (MOI 0.01). Cells were collected and processed 0–96 h post-infection for quantitation of viral RNA using RT-qPCR. Results represent the mean ± standard deviation (*n* = 3).

MDBK appeared to have lost one of its *GRID2* alleles prior to selection of the CRIB cell line (Allele 1 deletion, Figure 3A). During selection, a nested deletion resulted in a homozygous deleted region on chromosome 6 in CRIB cells that fell entirely within a non-coding region of *GRID2* (intron 8, Figure 3A). However, intronic deletions may be associated with variability in gene expression [36]; thus, mRNA transcript abundance was compared between MDBK and CRIB cells. *GRID2* mRNA expression was reduced by 50% in CRIB cells compared to MDBK cells (Figure 3B). Evaluation of GRID2 protein levels by immunoblot revealed GRID2 protein expression was also consistently reduced in CRIB cells compared to MDBK cells (Figure 3C). To determine whether altered *GRID2* expression impacts virus susceptibility in CRIB cells, MDBK *GRID2* KO clones were generated by introducing a premature termination codon and frameshift mutation in exon 6 (Figure 3A). The *GRID2* KO was confirmed by Sanger and WGS comparisons and by an anti-GRID2 immunoblot (Figure 3C). Infection of *GRID2* KO clones revealed that loss of *GRID2* did not significantly impact viral replication kinetics in MDBK cells (Figure 3D). Therefore, it is unlikely that the reduction in *GRID2* expression alone contributes to the BVDV resistant phenotype in CRIB cells.

### 3.4. Disruption of the RABGAP1L Gene Does Not Significantly Impact BVDV Infection in MDBK Cells

**Figure 4:**
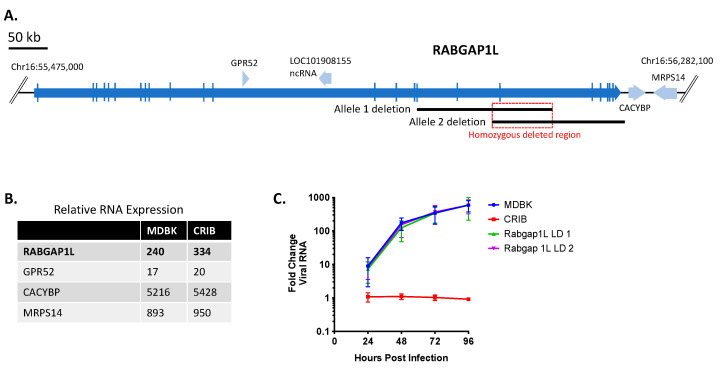
Disruption of the *RABGAP1L* gene does not significantly impact BVDV infection in MDBK cells. Panel (**A**), map of bovine *RABGAP1L* and surrounding genes: blue arrows, genes (open reading frames); black lines, heterozygous deletions in two alleles of *RABGAP1L* on chromosome 16; light red deletion block, homozygous deleted region (69,384 bp); red block, CRISPR/Cas9-mediated homozygous deletion. Shown is the corrected annotation for RABGAP1L based on IsoSeq data (see methods and Figure 1C). Panel (**B**), Relative RNA expression levels in MDBK and CRIB cells (TMM normalization value). Panel (**C**), Multistep virus growth curve. Cells were infected with BVDV strain NADL (MOI 0.01). Cells were collected and processed 0–96 h post-infection for quantitation of viral RNA using RT-qPCR. Results represent the mean ± standard deviation (*n* = 3).

The homozygous deleted region on chromosome 16 is predicted to remove exon 19 from both alleles encoding the *RABGAP1L* gene (Figure 4A). Moreover, each allele is missing additional exons from non-overlapping deletions such that neither allele would be expected to produce a functional full-length protein. The combined deletions did not substantially alter the abundance of RNA transcripts produced from this gene in CRIB cells (Figure 4B). However, the incorrect annotation of this gene in the reference genome used to quantify transcript abundance raises questions about the accuracy of this measurement. Therefore, due to annotation inaccuracies, complexities of the *RABGAP1L* isoform variants (Figure 1C), and a lack of commercial antibodies available to measure bovine RABGAP1L protein expression, we chose to make a 69 kb homozygous deletion in MDBK cells encompassing the entire haploid region found in CRIB cells (red box in Figure 4A). The homozygous deletion was confirmed by Sanger sequencing and WGS comparisons. However, deletion of this region of chromosome 16 did not significantly impact viral replication kinetics in MDBK cells (Figure 4C). Therefore, disruption of the RABGAP1L gene alone did not replicate the BVDV-resistant phenotype of CRIB cells.

### 3.5. Triple KO of PTPN12, GRID2, and RABGAP1L Genes Does Not Impact BVDV Infection in MDBK Cells

**Figure 5:**
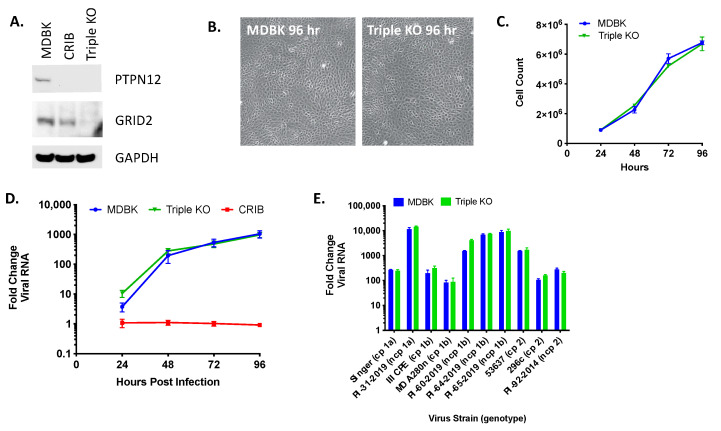
Triple KO of *PTPN12*, *GRID2*, and *RABGAP1L* genes does not impact BVDV infection in MDBK cells. Panel (**A**) western blot of SDS-PAGE for PTPN12, GRID2, and GAPDH (loading control). Panel (**B**) MDBK and MDBK-TKO cell morphology (10x). Panel (**C**) Cell growth in T-25 flasks. Panel (**D**) Multistep virus growth curve. Cells were infected with BVDV strain NADL (MOI 0.01). Cells were collected and processed 0–96 h post-infection for quantitation of viral RNA using RT-qPCR. Results represent the mean ± standard deviation (*n* = 3). Panel (**E**) Cells were infected with various cytopathic (cp) and non-cytopathic (ncp) BVDV isolates (MOI = 0.01) and collected at 0 and 96 h post-infection for quantitation of viral RNA using RT-qPCR. Results represent means at 96 h ± standard deviation (*n* = 3).

Individually, disruption of the three genes impacted by the three largest deletions in the CRIB cell line appeared to have no effect on viral entry or replication, but the experiments did not rule out the possibility that an interaction between the deletions might produce the observed resistance to viral infection. Therefore, triple KO clones were generated by transfecting the *RABGAP1L* KO cells with gRNAs and ssODN for PT*PN12* and *GRID2*. The efficiency of the triple KO was low and often led to a change in cell morphology and growth (data not shown). As a result, only one single-cell derived clone was generated for downstream testing. The protein expression levels of *PTPN12* and *GRID2* genes were assessed in this clone by immunoblot (Figure 5A) and all three mutations were confirmed by Sanger and WGS comparisons. The triple KO cells were similar in morphology but appeared slightly larger in size than MDBK cells (Figure 5B) and grew with similar kinetics (Figure 5C). However, loss of *GRID2*, *PTPN12*, and *RABGAP1L* did not affect viral replication kinetics when infected with various BVDV isolates (Figure 5D,E).

## 4. Discussion

Comparative genome analyses of BVDV-susceptible MDBK cells and BVDV-resistant CRIB cells were completed with the goal of identifying novel host factors required for BVDV entry. We identified three large homozygous deleted regions impacting genes on chromosomes 4, 6 and 16 that represented obvious viable candidates for affecting viral susceptibility. However, knocking out these genes individually or in combination did not reproduce the CRIB phenotype and suggests these genes are not necessary for BVDV entry or replication in the MDBK cell line.

The strength of this study was the ability to use genome wide analyses and gene editing to target the disruption of candidate genes in the parent MDBK cell line in order to directly test hypotheses of gene function. We used the CRISPR/Cas 9 system consisting of a single polypeptide endonuclease Cas9 complexed with a guide RNA (gRNA) to KO target genes in the MDBK cell line. This technology has been used to create precise mutations in target genes [37,38] to evaluate function of putative disease resistance alleles and improve animal welfare [39,40,41,42]. However, this study also had two main weaknesses. First, the KO strategy employed for two of the three genes (*PTPN12* and *GRID2*) removed activity of the target genes but did not precisely recreate the same deletion observed in CRIB cells. Therefore, we were only able to assess the requirement of the genes directly impacted by these deletions. It is possible that these large deletions disrupt the regulation of other genes or non-coding RNAs, thus impacting virus susceptibility. On the other hand, the KO strategy for *RABGAP1L* included a large region spanning the entire haploid region identified in CRIB cells. Thus, the large deletion impacting the coding region of *RABGAP1L* can be ruled out as the causative mutation leading to pestivirus resistance in CRIB cells.

The second weakness was limiting the focus to large deletions resulting in homozygous deleted genome segments. It is possible that compound deletions that do not overlap could still eliminate gene function and be involved in the phenotype. In addition, other variants including small deletions affecting coding regions; missense, nonsense, and frameshift mutations; and mutations affecting splice sites could underlie the resistance phenotype. There are currently only a few variant calling algorithms used for detecting differences between closely related genomes and it is not clear how best to prioritize their output [43,44,45]. Furthermore, technical challenges related to reference genome quality, annotation methods, sequencing artifacts, variation in read coverage depth, and sequence repeats make it difficult to detect true functional variants from spurious sequence differences and assembly errors. Various filters can be applied to the data; however, results can differ substantially between filtering methods. These technical limitations make smaller functional variants difficult to detect. Ideally, future work could use a combination of computational strategies, short- and long-read RNA sequencing data, and methods to detect epigenetic changes [46] in effort to help identify and rank variants to be evaluated.

Isolating additional BVDV-resistant MDBK clones may further aid in the identification of genes or biological pathways essential for BVDV infection. In our study, the true functional change leading to resistance may have been obscured by lack of access to the exact parental MDBK cell line and passage number used to select the resistant CRIB clone as well as the fact that the CRIB-1 clone obtained from ATCC for sequencing was at a relatively high passage (passage 37). Thus, there could have been genetic drift in both the MDBK and CRIB cell lines from the accumulation of additional changes (chromosomal rearrangements or point mutations) through continued passages. Whole genome sequencing of matched parental MDBK and new, independently selected BVDV-resistant CRIB clones may therefore reduce the number of variants detected between cells for downstream evaluation.

## 5. Conclusions

The changes that led to the development of the resistant phenotype in CRIB cells remain to be determined but are likely to be classified as either genetic or epigenetic perturbations to the cellular system. Although the experiments described here did not identify these changes, they tested the three observable, large deletions that have accrued in the CRIB cell line relative to the MDBK cell line, and we report them in detail to serve as a guide for future studies. More complex analyses of smaller deletions, insertions, and loss or gain of function mutations combined with global RNA expression data will be needed to reveal and rank additional candidate genes that may play a role in BVDV resistance. Generation and characterization of additional BVDV-resistant CRIB clones may also aid in the identification of genes, epigenetic factors, and biological pathways essential for pestivirus entry. Successful identification of such factors will significantly contribute to our understanding of host, tissue, and cell tropism and further delineate the complex nature of BVDV pathogenesis. In addition, it could reveal additional host targets for novel intervention strategies.

## Figures and Tables

**Table 1:** Guide RNAs and single-stranded oligo donors used in gene-editing.

Gene	gRNA(s) (5′-3′)	ssODN (5′-3′)
***PTPN12***	GAUUUUUGGAGGAUGAUAU	GACCTTTAGCAAATACGGTAATAGATTTTTGGAGGATGATATAAAGCTTTGGGAGTACAATGTTGTAGTAAGTATTGTATGAAATGGCAT
***GRID2***	GGAUCCAUUUGCUCAGAAUA	TATCTTCAACATTGTGTGATCCAAAGGATCCATTTGCTCAGTAAAGCTTAATATGGAGGTATATTCTAAGCACCCAGATATTTCTCTAAG
***RABGAP1L***	Targeting 5′: GGGAUUAUGCAGAAGAGGUTargeting 3′: GGAAUGUCACGUAGGCCUA	CACAAGAAATTATTAAGAGTAATTTTGGGATTATGCAGAAGAAAGCTTCTATGGCCTATCCTTCCATGCAACCAATAATTCTCTTTCCCT

Abbreviations: gRNA, guide RNA; ssODN, single-stranded oligo donor.

**Table 2:** Gene functions.

Gene	Cellular Localization and Expression	Gene Function
*PTPN12*	Cytosol; ubiquitously expressed	Member of the protein tyrosine phosphatase (PTP) family; plays role in cytoskeletal structure, cell adhesion, cell shape and mobility, and cell-cell junctions [32]
*GRID2*	Plasma membrane; predominantly expressed in the cerebellum	Ionotropic glutamate receptor in the mammalian brain; plays a role in synaptogenesis, synaptic plasticity, and endocytosis of the AMPA receptor [33].
*RABGAP1L*	Cytoplasm, endosome; ubiquitously expressed	Rab GTPase activating protein 1 like; plays a role in endocytosis, endosome maturation, and autophagosome formation [34,35].

## Data Availability

The datasets produced during this study are available in the NCBI BioProject repository under accession PRJNA761701 (MDBK, CRIB, and MDBK knockout clone WGS and MDBK and CRIB RNASeq).

## Supplementary Materials

The following are available online at https://www.mdpi.com/article/10.3390/v13112147/s1, Table S1. Gene names and ARS-UCD1.2 coordinates for the top three homozygous deletions in MDBK-CRIB cell line comparison. Figure S1. Integrative Genomics Viewer session showing MDBK+_CRIB- BED file.
