# Supplementary material for: Evaluating Large Spontaneous Deletions in a Bovine Cell Line Selected for Bovine Viral Diarrhea Virus Resistance
Source: Viruses. 2021 Oct 25;13(11):2147. doi: 10.3390/v13112147 (PMC8622392; doi:10.3390/v13112147)

Table S1. Gene names and ARS-UCD1.2 coordinates for the top three homozygous deletions in MDBK-CRIB cell line comparison.

Affected Genes (Chr)	MDBK		CRIB			
	Haploid region	Size (bp)	Haploid region	Size (bp)	Deleted region	Size (bp)
PTPN12 (4)	na	na	43,526,464<br>43,673,345	146,882	43,615,698<br>43,649,505	33,808
GRID2 (6)	31,032,548<br>31,504,974	476,785	31,032,548<br>31,504,974	472,427	31,216,568<br>31,279,767	63,200
RABGAP1L (16)	na	na	55,960,038<br>56,221,837	261,800	56,060,044<br>56,129,427	69,384

Abbreviations: Chr, chromosome; bp, base pairs

Figure S1. Integrative Genomics Viewer session showing MDBK+_CRIB- BED file. The BED file of MDBK cell reads mapping to regions of the genome not covered by reads from CRIB (BED file named “MDBK+_CRIB-.bed” marked with red *) was manually screened with the Integrative Genomics Viewer (IGV). The red arrows mark the three regions in the reference genome where there were a significant number of reads from MDBK mapping to the repeat masked reference bovine genome and no reads from the CRIB. These denote the regions of the CRIB genome containing compound homozygous deletions.

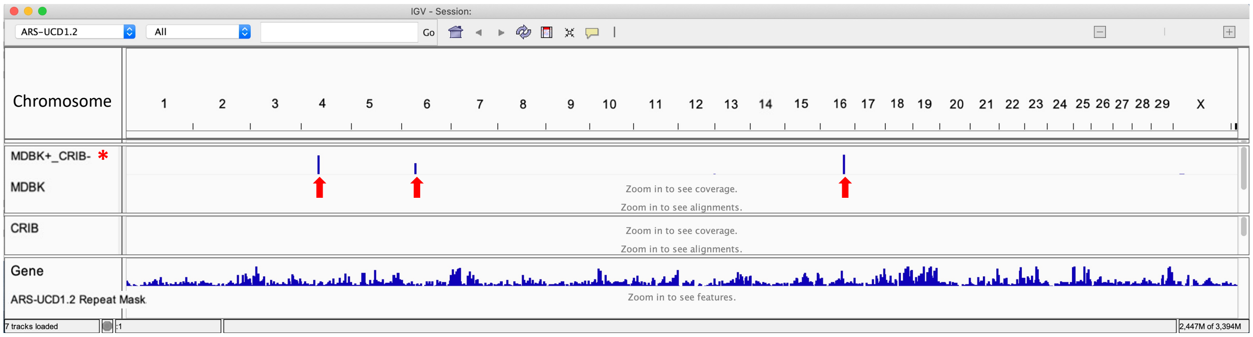

Supplement: Supplementary file 1 [file viruses-13-02147-s001.zip › viruses-1412056-supplementary.pdf]
